# Triglyceride-glucose index prediction of stroke incidence risk in low-income Chinese population: a 10-year prospective cohort study

**DOI:** 10.3389/fendo.2024.1444030

**Published:** 2024-10-17

**Authors:** Xiao Li, Juan Hao, Qingqing Han, Di Wang, Yuting Lu, Jun Tu, Lifeng Wang, Jinghua Wang, Xianjia Ning, Chunsheng Yang, Yan Li

**Affiliations:** ^1^ Department of Neurology, Tianjin Medical University General Hospital, Tianjin, China; ^2^ College of Anesthesiology, Shanxi Medical University, Taiyuan, China; ^3^ School of Basic Medical Sciences, Tianjin Medical University, Tianjin, China; ^4^ Laboratory of Epidemiology, Tianjin Neurological Institute, Tianjin, China; ^5^ Tianjin Neurological Institute, Key Laboratory of Post-Neuroinjury Neuro-repair and Regeneration in Central Nervous System, Ministry of Education and Tianjin City, Tianjin, China; ^6^ Institute of Clinical Epidemiology & Evidence-Based Medicine, Tianjin Jizhou People’s Hospital, Tianjin, China; ^7^ Department of Anesthesiology, Tianjin Jizhou People’s Hospital, Tianjin, China

**Keywords:** TyG index, stroke, risk factors, epidemiology, metabolism

## Abstract

**Aim:**

The Triglyceride-Glucose (TyG) index, an indicator of insulin resistance, has been proposed as a predictor of cardiovascular diseases. However, its role in predicting stroke risk, particularly in low-income populations, is not well understood. This study aimed to investigate the predictive value of the TyG index for stroke incidence in a low-income Chinese population, with a focus on gender and age-specific differences.

**Methods:**

This 10-year prospective cohort study included 3,534 participants aged ≥45 years from rural areas in northern China. Baseline data on demographic characteristics, lifestyle factors, and clinical measurements were collected. Participants were followed for stroke incidence, categorized into ischemic and hemorrhagic stroke. Multivariate logistic regression models were used to assess the association between the TyG index and stroke incidence, adjusting for potential confounders.

**Results:**

During the follow-up period, 368 participants (10.4%) experienced a stroke, with 327 ischemic and 31 hemorrhagic strokes. TyG index was significantly associated with total and ischemic stroke incidence but not hemorrhagic stroke. After adjusting for confounding factors, for every one standard deviation increase in TyG index, the risk of stroke increased by 32% for overall stroke (RR: 1.32; 95% CI: 1.08-1.61; P=0.006) and 39% for ischemic stroke (RR: 1.39; 95% CI: 1.12-1.73; P=0.003). The risk of stroke in the highest TyG tertile levels (tertile 3) increased by 49% (RR: 1.49; 95% CI 1.11-1.99; P=0.007) for overall stroke, compared to those in the lowest tertile levels (tertile 1). For ischemic stroke, the risk of stroke increased by 53% (RR: 1.53; 95% CI 1.12-2.11; P=0.008) in the highest TyG tertile levels (tertile 3) compared to those in the lowest tertile levels (tertile 1).

**Conclusion:**

This 10-year prospective cohort study has established the TyG index as an independent predictor of both total and ischemic stroke incidence in a low-income Chinese population. The findings indicate that the TyG index is particularly effective in predicting stroke risk among women and older adults (≥60 years), but not for hemorrhagic stroke. These insights are crucial for improving clinical practice and stroke prevention strategies.

## Introduction

Stroke is the second leading cause of disability and death worldwide, particularly affecting individuals over 50 years old, with ischemic stroke being the most common subtype, accounting for about 80% of cases (1). In China alone, there were 3.94 million new stroke cases in 2019, marking an 86.0% increase in incidence since 1990, reaching 276.7 cases per 100,000 people (2). This trend is expected to continue, with the American Heart Association projecting that 3.4 million U.S. adults will have a stroke by 2030, a 20.5% increase from 2012 (3). This growing burden underscores the critical need for early identification of stroke risk factors and targeted prevention strategies.

Recent advancements in stroke research have identified various risk factors and biomarkers. One such biomarker, the TyG index, was first proposed in 2008 as a simple, convenient, and low-cost alternative to assess insulin resistance (IR) (4). Elevated TyG index levels have been independently associated with an increased risk of cardiovascular diseases (CVD) and mortality (5–8). Additionally, emerging evidence suggests a link between higher TyG index levels and an increased risk of cerebrovascular diseases, including ischemic stroke (9). However, most studies have focused on urban populations, leaving a gap in understanding its impact on low-income rural populations.

Despite these advancements, there remains considerable debate regarding the TyG index’s predictive value for different stroke subtypes. Some studies suggest a strong association between elevated TyG index and ischemic stroke but not hemorrhagic stroke (10). Moreover, the lack of research focusing on low-income rural populations, who may have different risk profiles and healthcare access, highlights a significant gap in the current literature.

While large-scale studies such as the PURE study by Lopez-Jaramillo et al. have extensively examined cardiovascular outcomes across diverse countries and income levels (5), specific attention to stroke risk in rural, low-income populations remains limited. This population faces unique challenges, such as limited healthcare access, low health literacy, and a higher prevalence of untreated cardiovascular risk factors, all of which can significantly contribute to stroke incidence. Therefore, the generalizability of findings from broader studies to these underrepresented groups may be limited.

Therefore, this study aims to investigate the predictive effect of the TyG index on stroke incidence risk in a low-income Chinese population. By focusing on this underrepresented group, we hope to provide insights that can inform targeted prevention strategies and ultimately reduce the stroke burden in similar communities.

## Methods

### Study design and population

This study was a 10-year prospective cohort study, with the baseline survey conducted from April to June 2014. All participants were recruited as part of the Tianjin Brain Study, a population-based surveillance program for cardiovascular and cerebrovascular diseases (11–13). The study population was drawn from 18 administrative villages, where approximately 95% of participants were low-income farmers, with a per capita disposable income of less than $1,600 in 2014 (14). Eligible participants were residents aged 45 years and older who had no prior history of cardiovascular or cerebrovascular diseases. The population was characterized by advanced age, low income, and limited education, as well as low levels of health awareness. All participants have been continuously followed since enrollment.

The study protocol was approved by the Tianjin Medical University General Hospital Ethics Committee (IRB2018-100-01), and all participants provided written informed consent.

### Definition and monitoring of stroke and CVD

From June 30, 2014, to December 31, 2023, all participants were followed to identify stroke events, CVD events, and all-cause mortality. A first stroke event was defined as the rapid onset of symptoms and signs of focal brain injury, lasting more than 24 hours, and confirmed by imaging evidence. Stroke events were identified through three sources: local licensed village doctors reporting according to a predetermined procedure, medical records for hospital inpatients, and the all-cause death registry.

CVD events included newly diagnosed coronary atherosclerotic heart disease, myocardial infarction, and cardiovascular death. Coronary atherosclerotic heart disease was defined as the presence of more than 50% narrowing in one or more coronary arteries, as demonstrated by coronary angiography.

Information on stroke and CVD events came from three sources as described previously (11, 13). Briefly, local licensed village doctors (LLVD) reporting according to a predetermined procedure, medical records for hospital inpatients, and the all causes of death registry.

### Risk factor assessment

Data on gender, age, and years of education were collected from existing records. Participants were categorized into three age groups (45-59 years, 60-74 years, and ≥75 years) and four education groups (illiterate, 1-6 years, 7-9 years, and ≥10 years). Smoking status was defined as smoking more than one cigarette per day for at least one year and categorized into never smoked, quit smoking, and current smoking. Alcohol consumption was defined as drinking more than 500 grams per week for at least one year and categorized into never drank, quit drinking, and current drinking. Additionally, data on participants’ history of atrial fibrillation and medication use, including lipid-lowering agents, antiplatelets, and anticoagulants, were collected through self-reported information.

### Clinical measurements and definitions

Systolic blood pressure (SBP) and diastolic blood pressure (DBP) were recorded as the average of two measurements. Hypertension was defined as SBP above 140 mmHg, DBP above 90 mmHg, or the use of antihypertensive drugs. Diabetes was defined as fasting blood glucose (FPG) of 7.0 mmol/L or higher, or the use of insulin or oral hypoglycemic agents. Body mass index (BMI) was calculated as weight (kg) divided by the square of height (m^2^) and categorized into normal weight (18.5 Kg/m^2^ ≤ BMI < 24.0 Kg/m^2^), overweight (24.0 Kg/m^2^≤ BMI < 28.0 Kg/m^2^), and obesity (BMI ≥ 28.0 Kg/m^2^).

### Biochemical measurements

Participants were instructed to fast from midnight (after 24:00) the day before blood collection. Fasting blood samples were collected at 8:00 a.m. the following morning, ensuring a fasting period of at least 8 hours. Measurements included complete blood count, FPG, triglycerides (TG), total cholesterol (TC), low-density lipoprotein cholesterol (LDL-C), and high-density lipoprotein cholesterol (HDL-C). The TyG index was calculated as Ln[TG (mg/dL) × FPG (mg/dL)/2] (4). All samples were processed in the central laboratory of Tianjin Jizhou People’s Hospital within 2 hours of collection.

### Statistical analyses

The normality of continuous variables was tested using the single-sample Kolmogorov-Smirnov test. For normally distributed continuous variables, the Student’s t-test was applied to compare group differences, with results expressed as mean (standard deviation). For non-normally distributed continuous variables, the rank-sum test was used, and results were expressed as medians (25th and 75th percentiles). Categorical variables were expressed as numbers (percentages), and the chi-square test was used to compare the distribution difference between groups.

To evaluate the predictive value of the TyG index for stroke onset, receiver operating characteristic (ROC) curves were generated, and the area under the curve (AUC) was calculated. Multivariate logistic regression analysis was employed to examine the association between stroke and its subtypes, with stroke onset as the dependent variable, the TyG index as the main independent variable, and other covariates selected based on statistical significance in the univariate analysis. The TyG index was analyzed both as a continuous variable and by tertile grouping to assess its relationship with stroke risk. Results were reported as adjusted relative risks (RR) with 95% confidence intervals (95% CI). Additionally, a restricted cubic spline (RCS) analysis was performed to explore potential non-linear relationships between the TyG index and stroke risk. All statistical analyses were conducted using IBM SPSS Statistics for Windows, version 25.0 (IBM Corp., Armonk, NY, USA) and R, version 4.2.3 (R Development Core Team, Vienna, Austria). A two-tailed p-value of <0.05 was considered statistically significant.

## Results

**Figure 1:**
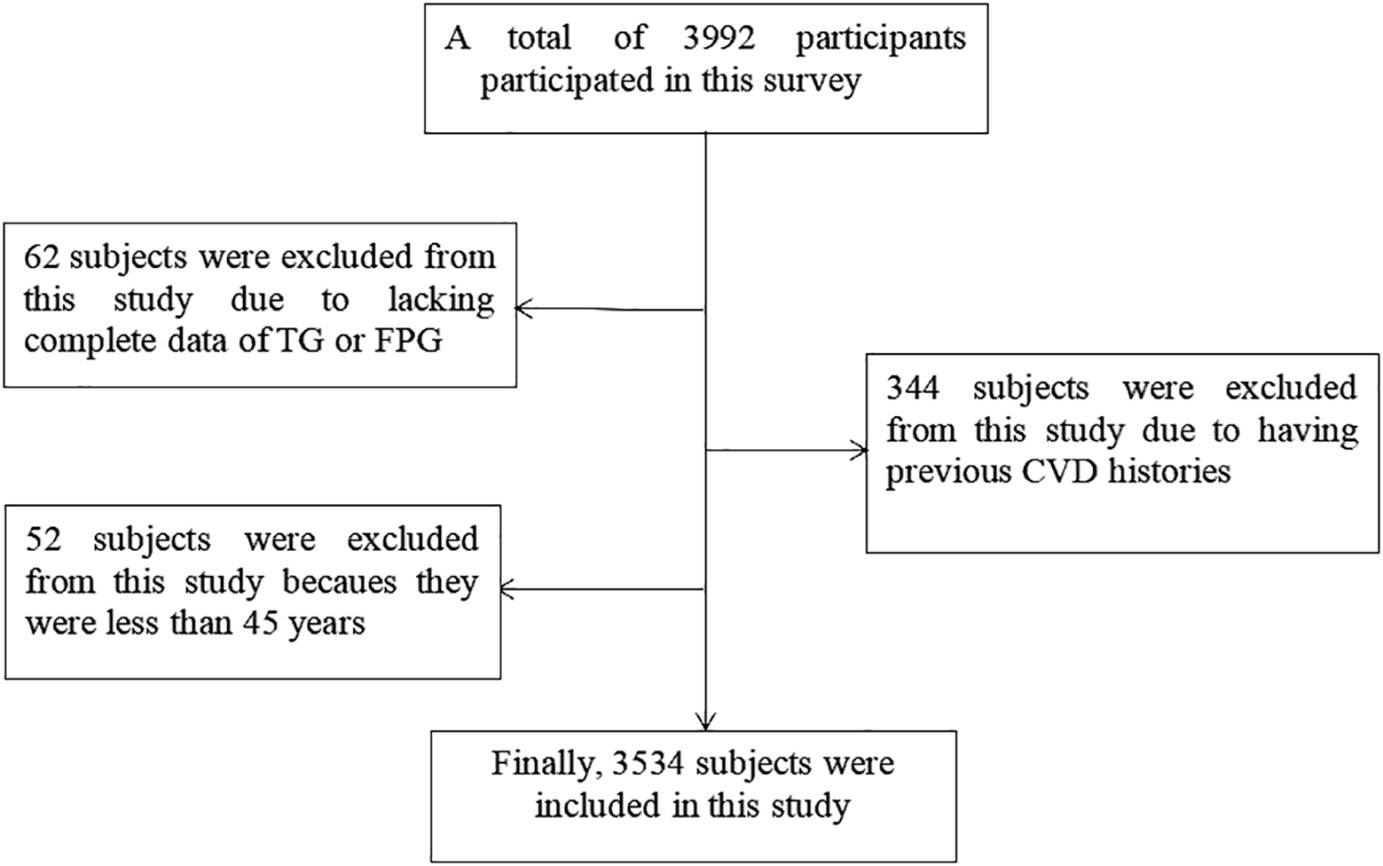
Flow chat of participants selection. Figure showed that total of 3992 participants were recruited during study periods, and 3648 participants met the inclusion criteria after excluded 344 participants with the previous CVD histories. Moreover, the basic data of TG or FPG in 62 participants was missing, and 52 participants were less than 45 years were excluded. Finally, 3534 participants were analyzed in this study.

A total of 3992 participants were recruited during study periods, and 3648 participants met the inclusion criteria after excluded 344 participants with the previous CVD histories. Moreover, the basic data of TG or FPG in 62 participants was missing, and 52 participants were less than 45 years were excluded. Finally, 3534 participants were analyzed in this study (**Figure 1**).

### Baseline characteristics

The study included 3,534 participants, of whom 1,419 (40.2%) were men and 2,115 (59.8%) were women. The median age of the participants was 59.05 years (25th percentile: 51.75, 75th percentile: 65.67), with a mean follow-up period of 9.04 years. Participants were divided into three groups based on tertiles of the TyG index: tertile 1 (TyG index < 8.51), tertile 2 (8.51 ≤ TyG index ≤ 9.04), and tertile 3 (TyG index > 9.04). A total of 19 participants (0.54%) self-reported taking oral lipid-lowering statins. None of the participants used antiplatelet drugs or anticoagulants, and no participant had a history of atrial fibrillation. Compared to those in the lowest tertile, participants in the higher tertiles were more likely to be female, obese, hypertensive, or diabetic, and less likely to have a history of smoking or alcohol consumption. Higher TyG index levels were associated with elevated SBP, DBP, LDL-C, TC, TG, and FPG levels, and lower HDL-C levels (**Table 1**).

**Table 1:** Baseline characteristics of the study population according to tertile groups of TyG index .

Characteristic	Total	TyG index			P
		Tertile 1	Tertile 2	Tertile 3	
Case, n (%)	3534 (100)	1177 (33.3)	1178 (33.3)	1179 (33.4)	
Gender, n (%)					<0.001
Men	1419 (40.2)	573 (40.4)	459 (32.3)	387 (27.3)	
Women	2115 (59.8)	604 (28.6)	719 (34.0)	792 (37.4)	
Age^*^, years	59.05 (51.75, 65.67)	59.24 (51.34, 66.07)	59.05 (52.12, 66.02)	58.92 (52.00, 65.04)	0.556
Age groups, n (%)					0.433
45-59 years	1925 (54.5)	620 (32.2)	643 (33.4)	662 (34.4)	
60-74 years	1326 (37.5)	456 (34.4)	437 (33.0)	433 (32.7)	
≥75 years	283 (8.0)	101 (35.7)	98 (34.6)	84 (29.7)	
Education groups, n (%)					0.229
0 year	678 (19.3)	208 (30.7)	224 (33.0)	246 (36.3)	
1-6 years	1512 (43.1)	526 (34.8)	517 (34.2)	469 (31.0)	
7-9 years	1070 (30.5)	354 (33.1)	344 (32.1)	372 (34.8)	
≥10 years	249 (7.1)	82 (32.9)	82 (32.9)	85 (34.1)	
BMI groups, n (%)					<0.001
Normal	1225 (34.7)	576 (47.0)	432 (35.3)	217 (17.7)	
Overweight	1486 (42.1)	442 (29.7)	498 (33.5)	546 (36.7)	
Obesity	817 (23.2)	156 (19.1)	246 (30.1)	415 (50.8)	
Smoking status, n (%)					<0.001
Current smoking	737 (20.9)	293 (39.8)	232 (31.5)	212 (28.8)	
Quit smoking	152 (4.3)	61 (40.1)	49 (32.2)	42 (27.6)	
Never smoked	2645 (74.8)	823 (31.1)	897 (33.9)	925 (35.0)	
Alcohol consumption, n (%)					0.007
Current drinking	510 (14.4)	196 (38.4)	174 (34.1)	140 (27.5)	
Quit drinking	40 (1.1)	18 (45.0)	10 (25.0)	12 (30.0)	
Never drank	2984 (84.4)	963 (32.3)	994 (33.3)	1027 (34.4)	
Hypertension, n (%)					<0.001
Yes	2442 (69.1)	705 (28.9)	822 (33.7)	915 (37.5)	
No	1091 (30.9)	472 (43.3)	355 (32.5)	264 (24.2)	
Diabetes, n (%)					<0.001
Yes	671 (19.0)	74 (11.0)	176 (26.2)	421 (62.7)	
No	2863 (81.0)	1103 (38.5)	1002 (35.0)	758 (26.5)	
Statins use, n (%)					0.367
Yes	19 (0.5)	6 (31.6)	4 (21.1)	9 (47.4)	
No	3515 (99.5)	1171 (33.3)	1174 (33.4)	1170 (33.3)	
SBP^*^,mmHg	144.00 (130.33, 160.00)	140.67 (127.67, 157.83)	144.00 (131.00, 159.50)	146.50 (133.00, 162.00)	<0.001
DBP^*^,mmHg	86.00 (79.00, 93.67)	84.00 (77.00, 91.00)	86.50 (79.00, 94.00)	88.00 (81.00, 95.50)	<0.001
Hb^*^, g/L	138.00 (129.00, 147.00)	138.00 (129.75, 148.00)	137.00 (129.00, 146.50)	138.00 (129.00, 147.00)	0.841
Plt^*^, 10^9^/L	231.00 (197.00, 271.00)	229.00 (198.00, 270.00)	231.00 (195.50, 272.00)	232.00 (197.00, 272.00)	0.925
FPG^*^, mmol/L	5.60 (5.10, 6.10)	5.30 (4.90, 5.65)	5.60 (5.17, 6.10)	5.93 (5.40, 7.10)	<0.001
TC^*^, mmol/L	4.79 (4.15, 5.50)	4.44 (3.86, 5.06)	4.75 (4.18, 5.44)	5.20 (4.56, 5.99)	<0.001
TG^*^, mmol/L	1.40 (1.01, 2.11)	0.89 (0.73, 1.04)	1.42 (1.25, 1.64)	2.55 (2.09, 3.36)	<0.001
HDL-C^*^, mmol/L	1.39 (1.15, 1.70)	1.54 (1.30, 1.84)	1.39 (1.16, 1.73)	1.25 (1.05, 1.49)	<0.001
LDL-C^*^, mmol/L	2.59 (2.04, 3.22)	2.44 (1.96, 3.00)	2.63 (2.10, 3.27)	2.72 (2.06, 3.39)	<0.001

^*^Continuous variables were expressed as medians (percentile25, percentile75). TyG index, triglyceride-glucose index; BMI, body mass index; SBP, systolic blood pressure; DBP, diastolic blood pressure; Hb, hemoglobin; Plt, platelet; FPG, fasting plasma glucose; TG, triglycerides; TC, total cholesterol; LDL-C, low-density lipoprotein cholesterol; HDL-C, high-density lipoprotein cholesterol.

### Incidence of stroke and CVD

During the 10-year follow-up period, a total of 368 participants (10.4%) experienced a new stroke, 32 participants (0.9%) were diagnosed with a new CVD and 406 participants (11.5%) died from all causes. Of these, 327 were ischemic strokes, 31 were hemorrhagic strokes, and 10 were unclassified strokes.

**Figure 2:**
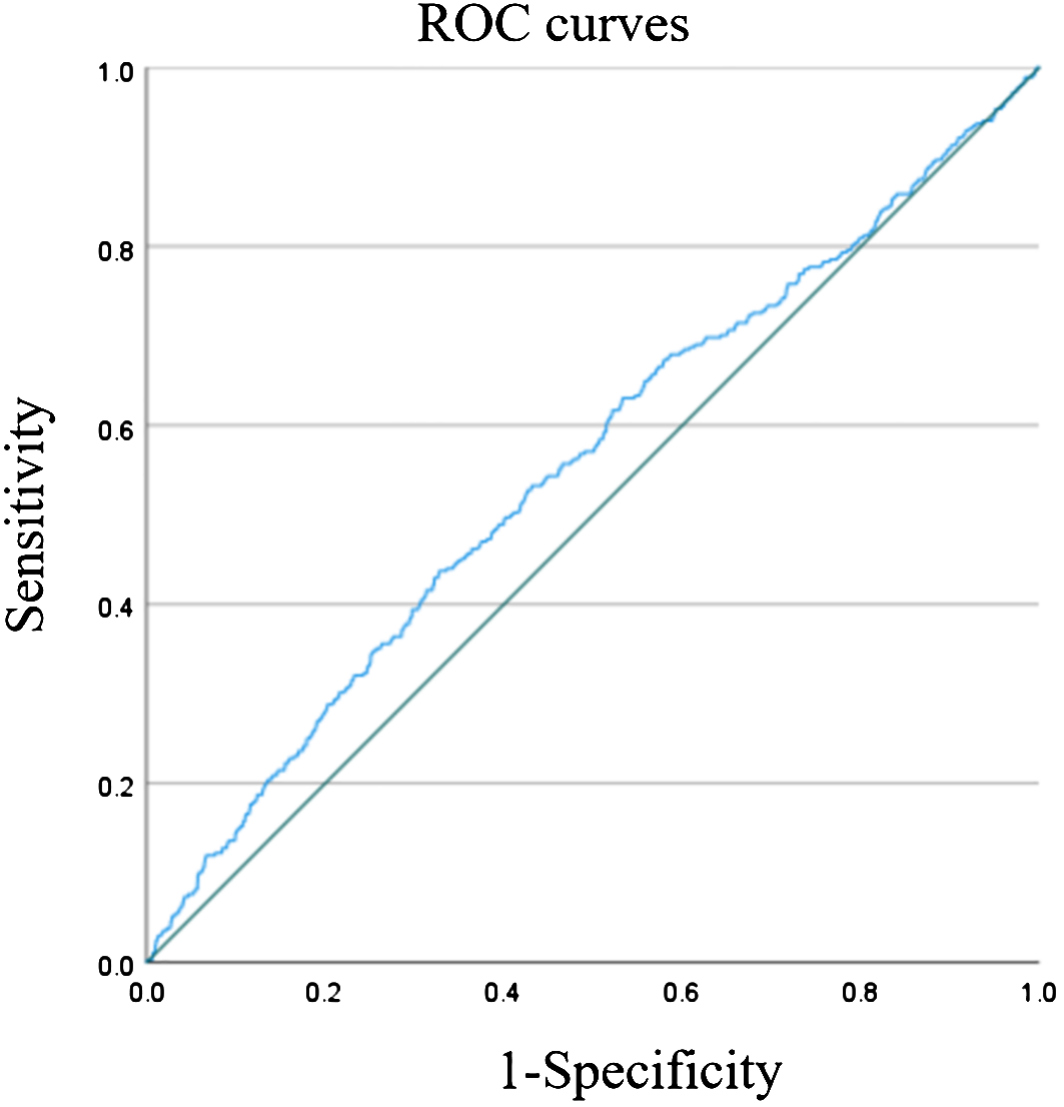
ROC curve for TyG index to predict stroke. Figure showed that the AUC of TyG index in predicting stroke was 0.554 (95% CI: 0.522-0.586; P<0.001). The cut-off point was 9.04. It was suggested that TyG index is an important predictor of stroke, and when TyG index > 9.04, the risk of stroke would increase.

The ROC curve for TyG index in predicting stroke incidence is presented in **Figure 2**. The AUC for the TyG index was 0.554 (95% CI: 0.522–0.586; P < 0.001), with a cut-off point of 9.04. These results suggest that the TyG index serves as an important predictor of stroke, with stroke risk increasing significantly when the TyG index exceeds 9.04.

### Association of TyG index with stroke incidence in the univariate study

In univariate analysis, several factors were significantly associated with stroke onset, including male gender, older age, smoking, hypertension, diabetes, higher SBP, DBP, FBG, TC, LDL-C, and TyG index (all P<0.05; **Table 2**). However, no significant association was observed between the TyG index and CVD incidence in the univariate analysis (P > 0.05; **Supplementary Table S1**).

**Table 2:** The associated factors of stroke onset in the study population by types in univariate analysis.

Characteristic	Stroke			Ischemic stroke			Hemorrhagic stroke		
	No	Yes	P	No	Yes	P	No	Yes	P
Case, n (%)	3166 (89.6)	368 (10.4)	—	3207 (90.7)	327 (9.3)	—	3503 (99.1)	31 (0.9)	—
Gender, n (%)			<0.001			<0.001			0.191
Men	1231 (86.8)	188 (13.2)		1252 (88.2)	167 (11.8)		1403 (98.9)	16 (1.1)	
Women	1935 (91.5)	180 (8.5)		1955 (92.4)	160 (7.6)		2100 (99.3)	15 (0.7)	
Age^*^, years	58.52 (51.42,64.99)	63.20(58.03,69.79)	<0.001	58.65 (51.47, 65.16)	62.82 (57.88, 68.83)	<0.001	59.01 (51.71, 65.63)	64.75 (57.53, 69.97)	0.008
Age groups, n (%)			<0.001			<0.001			0.058
45-59 years	1800 (93.5)	125 (6.5)		1811 (94.1)	114 (5.9)		1914 (99.4)	11 (0.6)	
60-74 years	1130 (85.2)	196 (14.8)		1153 (87.0)	173 (13.0)		1308 (98.6)	18 (1.4)	
≥75 years	236 (83.4)	47 (16.6)		243 (85.9)	40 (14.1)		281 (99.3)	2 (0.7)	
BMI groups, n (%)			0.057			0.030			0.928
Normal	1114 (90.9)	111 (9.1)		1128 (92.1)	97 (7.9)		1215 (99.2)	10 (0.8)	
Overweight	1330 (89.5)	156 (10.5)		1349 (90.8)	137 (9.2)		1473 (99.1)	13 (0.9)	
Obesity	716 (87.6)	101 (12.4)		724 (88.6)	93 (11.4)		809 (99.0)	8 (1.0)	
Smoking status, n (%)			<0.001			<0.001			0.289
Current smoking	633 (85.9)	104 (14.1)		645 (87.5)	92 (12.5)		727 (98.6)	10 (1.4)	
Quit smoking	131 (86.2)	21 (13.8)		132 (86.8)	20 (13.2)		151 (99.3)	1 (0.7)	
Never smoked	2402 (90.8)	243 (9.2)		2430 (91.9)	215 (8.1)		2625 (99.2)	20 (0.8)	
Alcohol consumption, n (%)			0.102			0.016			0.808
Current drinking	445 (87.3)	65 (12.7)		450 (88.2)	60 (11.8)		506 (99.2)	4 (0.8)	
Quit drinking	38 (95.0)	2 (5.0)		40 (100)	0 (0)				
Never drank	2683 (89.9)	301 (10.1)		2717 (91.1)	267 (8.9)		2997 (99.1)	27(0.9)	
Hypertension, n (%)			<0.001			<0.001			<0.001
Yes	2111 (86.4)	331 (13.6)		2149 (88.0)	294 (12.0)		2412 (98.8)	30 (1.2)	
No	1054 (96.6)	37 (3.4)		1058 (97.0)	33 (3.0)		1090 (99.9)	1 (0.1)	
Diabetes, n (%)			<0.001			<0.001			0.058
Yes	550 (82.0)	121 (18.0)		561 (83.6)	110 (16.4)		661 (98.5)	10 (1.5)	
No	2616 (91.4)	247 (8.6)		2646 (92.4)	217 (7.6)		2842 (99.3)	21 (0.7)	
SBP^*^,mmHg	142.50 (129.00, 158.50)	155.00 (142.00, 173.00)	<0.001	142.50 (129.50, 158.50)	154.33 (141.50, 173.00)	<0.001	143.50 (130.00, 160.00)	155.50 (145.50, 173.00)	<0.001
DBP^*^,mmHg	85.50(78.50, 93.00)	91.33(83.13,99.00)	<0.001	85.50 (78.67, 93.00)	91.00 (83.50, 99.00)	<0.001	86.00 (79.00, 93.67)	91.67 (81.50, 97.00)	0.014
Hb*, g/L	137.00 (129.00, 147.00)	138.00 (130.00, 147.75)	0.356	137.00 (129.00, 147.00)	138.00 (130.00, 148.00)	0.270	137.50 (129.00, 147.00)	139.50 (128.75, 147.00)	0.791
Plt^*^, 10^9^/L	231.00 (197.00, 273.00)	223.00 (200.00, 258.50)	0.255	231.00 (197.00, 272.00)	223.00 (198.00, 260.00)	0.285	231.00 (197.00, 271.75)	223.00 (200.25, 259.75)	0.557
FPG^*^, mmol/L	5.56 (5.10, 6.10)	5.80 (5.30, 6.70)	<0.001	5.56 (5.10, 6.10)	5.80 (5.36, 6.77)	<0.001	5.60 (5.10, 6.10)	5.60 (5.23, 6.20)	0.650
TC^*^, mmol/L	4.77 (4.13, 5.48)	4.95 (4.27, 5.77)	<0.001	4.77 (4.14, 5.49 )	4.94 (4.27, 5.76)	0.002	4.79 (4.15, 5.50)	5.30 (4.24, 6.07)	0.096
TG^*^, mmol/L	1.39 (1.01, 2.09)	1.52 (1.01, 2.21)	0.151	1.39 (1.01, 2.09)	1.52 (1.04, 2.21)	0.054	1.40 (1.01, 2.11)	1.39 (0.81, 1.92)	0.402
HDL-C^*^, mmol/L	1.39 (1.15, 1.70)	1.38 (1.14, 1.67)	0.529	1.39 (1.15, 1.70)	1.36 (1.14, 1.67)	0.430	1.39 (1.15, 1.70)	1.42 (1.13, 1.70)	0.624
LDL-C^*^, mmol/L	2.57 (2.02, 3.20)	2.71 (2.21, 3.37)	0.001	2.58 (2.03, 3.20)	2.70 (2.20, 3.38)	0.007	2.59 (2.04, 3.21)	2.75 (2.20, 3.38)	0.312
TyG index^*^	8.75 (8.38, 9.20)	8.88 (8.44, 9.37)	0.001	8.75 (8.38, 9.20)	8.91 (8.45, 9.40)	<0.001	8.77 (8.39, 9.22)	8.73 (8.18, 9.30)	0.560
TyG index tertile groups,n (%)			<0.001			<0.001			0.401
Tertile 1	1072 (91.1)	105 (8.9)		1088 (92.4)	89 (7.6)		1164 (98.9)	13 (1.1)	
Tertile 2	1073 (91.1)	105 (8.9)		1084 (92.0)	94 (8.0)		1171 (99.4)	7 (0.6)	
Tertile 3	1021 (86.6)	158 (13.4)		1035 (87.8)	144 (12.2)		1168 (99.1)	11 (0.9)	

^*^Continuous variables were expressed as medians (percentile25, percentile75). BMI, body mass index; SBP, systolic blood pressure; DBP, diastolic blood pressure; Hb, hemoglobin; Plt, platelet; FPG, fasting plasma glucose; TG, triglycerides; TC, total cholesterol; LDL-C, low-density lipoprotein cholesterol; HDL-C, high-density lipoprotein cholesterol; TyG index, triglyceride-glucose.

### Association of TyG index with stroke incidence in the multivariate study

TyG index was significantly associated with total and ischemic stroke incidence but not hemorrhagic stroke. After adjusting for confounding factors, for every one standard deviation increase in TyG index, the risk of stroke increased by 32% for overall stroke (RR: 1.32; 95% CI: 1.08-1.61; P=0.006) and 39% for ischemic stroke (RR: 1.39; 95% CI: 1.12-1.73; P=0.003).

**Figure 3:**
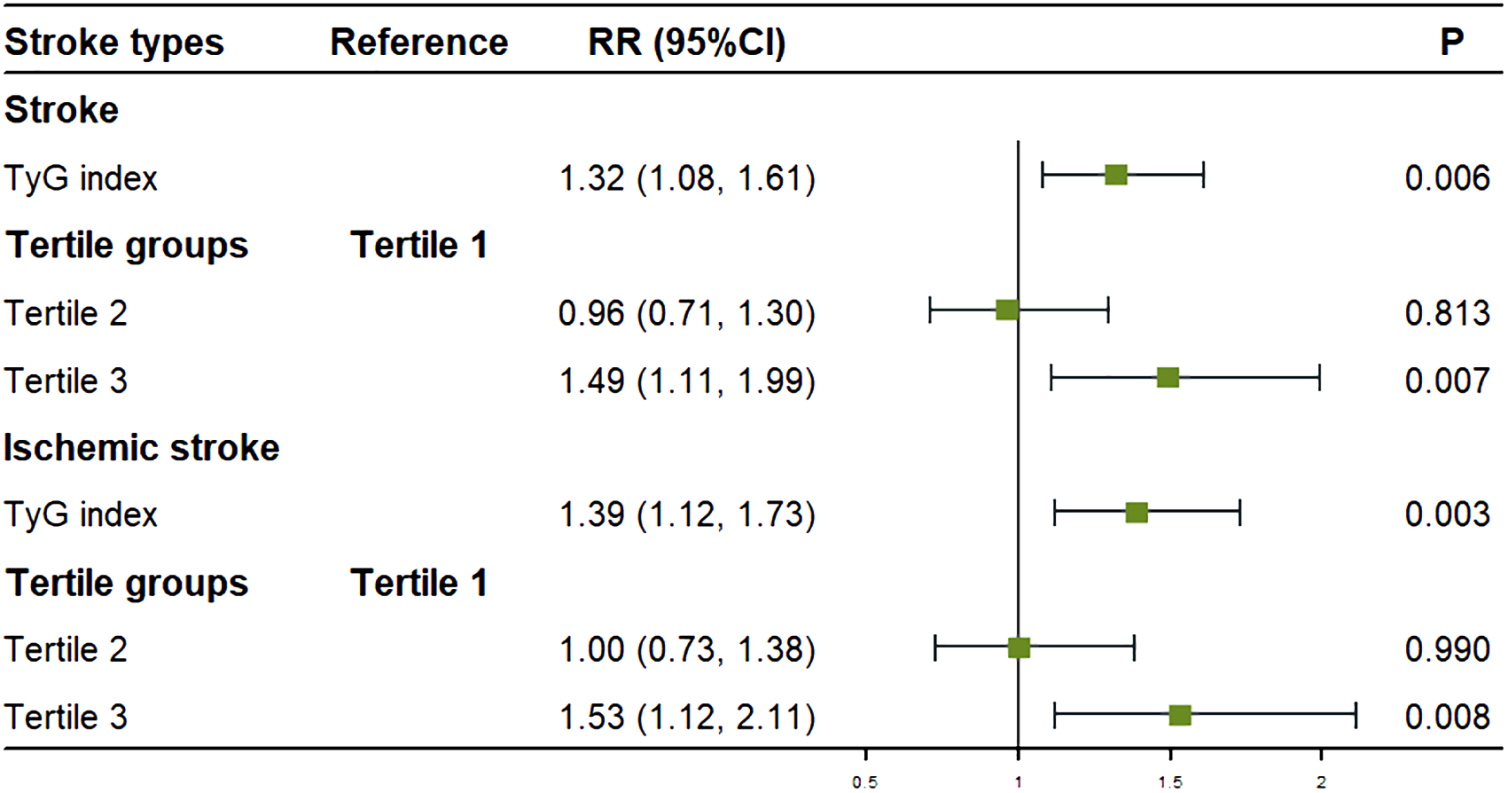
Association between the TyG index and stroke onset in multivariate analysis. Figure showed that after adjusting for covariates, for every one standard deviation increase in TyG index, the risk of stroke increased by 32% for overall stroke and 39% for ischemic stroke. Compared to tertile 1, the risk of stroke was increased by 49% for overall stroke and 53% for ischemic stroke in tertile 3.

After adjusting for sex, age group, smoking status, LDL-C, and history of hypertension, participants in the highest TyG tertile (tertile 3) had a 49% higher risk of overall stroke (RR: 1.49; 95% CI: 1.11–1.99; P = 0.007) compared to those in the lowest tertile (tertile 1). Similarly, after adjusting for sex, age group, BMI, smoking status, LDL-C, alcohol consumption, and history of hypertension, participants in the highest TyG tertile (tertile 3) had a 53% higher risk of ischemic stroke (RR: 1.53; 95% CI: 1.12–2.11; P = 0.008) compared to those in the lowest tertile. No significant association was found between the TyG index and hemorrhagic stroke incidence (**Figure 3**).

### Association of TyG index with stroke incidence in the subgroup analysis

Further analysis was stratified by sex and age, with the univariate results presented in **Supplementary Tables S2**, **S3**.

The multivariate analysis revealed that among women, after adjusting for age, BMI groups, LDL-C, and history of hypertension, every one standard deviation increase in the TyG index was associated with a 76% higher risk of stroke (RR: 1.76; 95% CI: 1.31–2.38; P < 0.001). Compared to participants in tertile 1, the risk of stroke was significantly increased by 90% (RR: 1.90; 95% CI: 1.21–3.00; P = 0.006) in tertile 3.

For participants aged ≥60 years, after adjusting for sex, LDL-C, and history of hypertension, every one standard deviation increase in the TyG index was associated with a 33% higher risk of stroke (RR: 1.33; 95% CI: 1.03–1.74; P = 0.028). In this age group, the risk of stroke was 59% higher in tertile 3 compared to tertile 1 (RR: 1.59; 95% CI: 1.11–2.29; P = 0.012).

**Figure 4:**
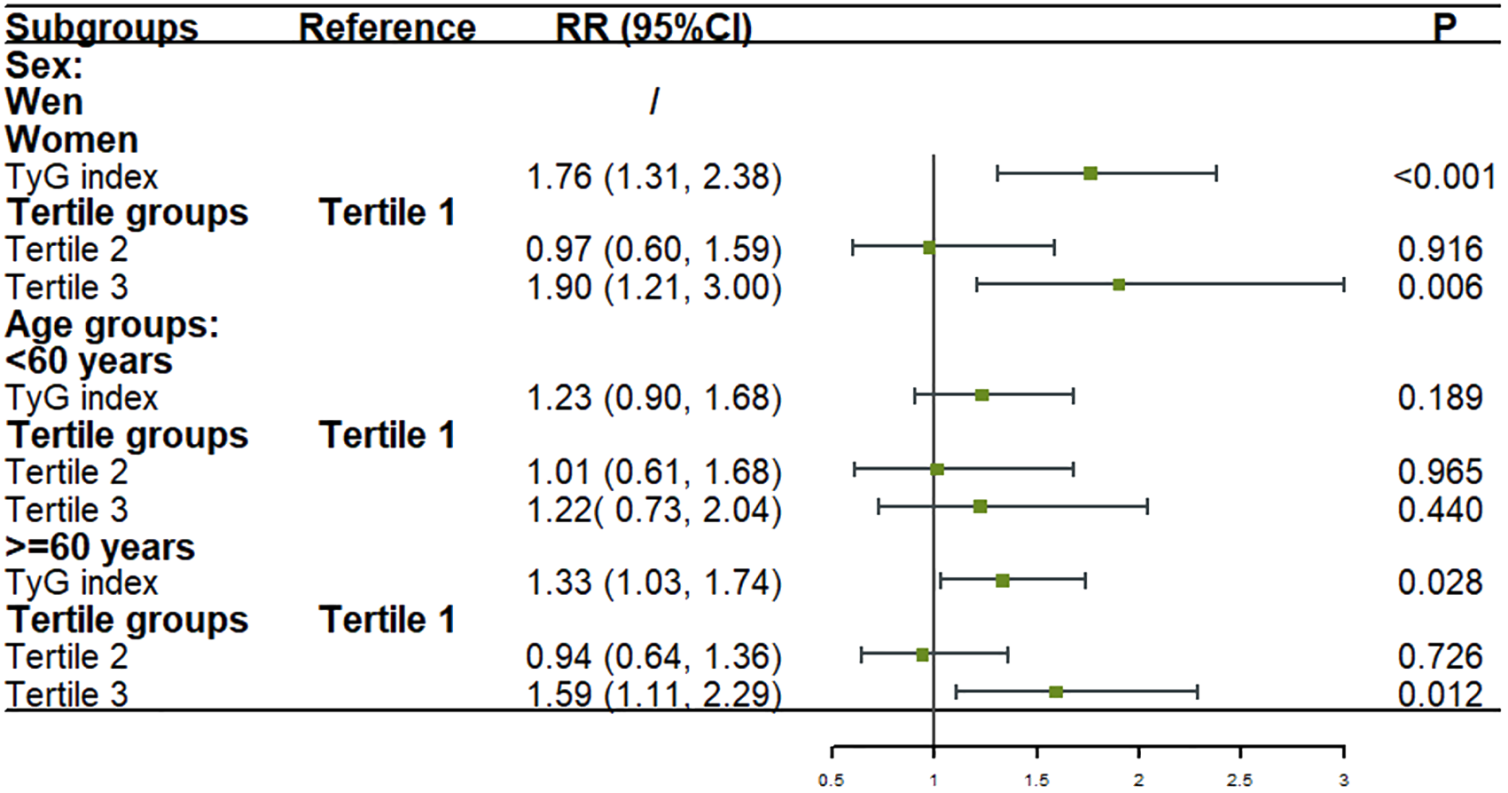
Association between the TyG index and stroke onset in sex and age subgroups in logistic models. Figure showed that after adjusting for covariates, for every one standard deviation increase in TyG index, the risk of stroke increased by 76% among women and 33% among participants aged ≥60 years. Compared to tertile 1, the risk of stroke was significantly increased by 90% among women and 59% among participants aged ≥60 years in tertile 3. No significant associations were found among men and younger participants after adjusting for confounding factors.

No significant associations were found among men and younger participants after adjusting for confounding factors (**Figure 4**).

### Non-linear relationship between TyG index and stroke incidence

**Figure 5:**
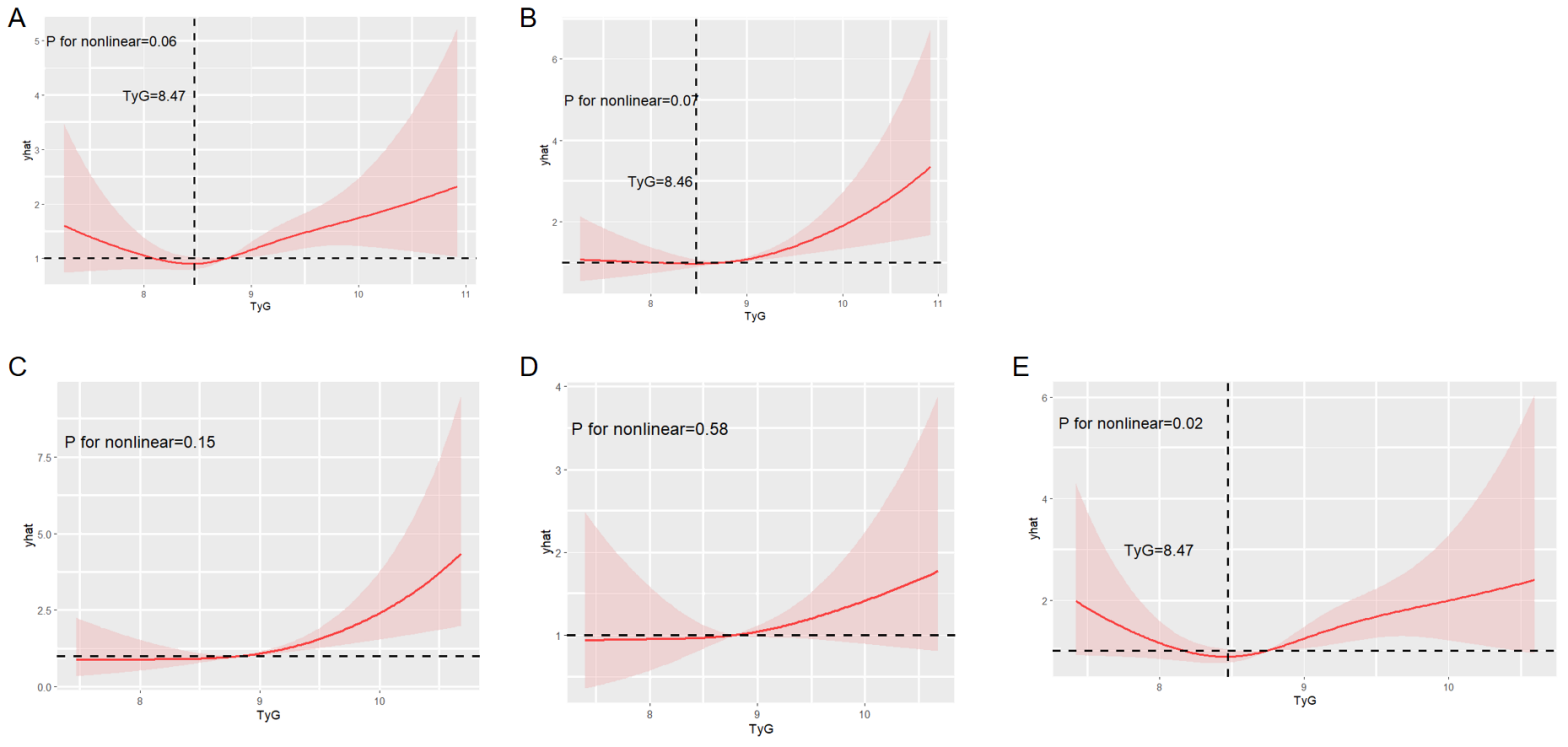
Restricted cubic spline analysis to evaluate the potential non-linear relationship between the TyG index and stroke incidence. Figure showed the potential non-linear relationship between overall stroke incidence **(A)**, ischemic stroke incidence **(B)**, stroke incidence in female subgroup **(C)**, stroke incidence in <60 years subgroup **(D)**, stroke incidence in ≥60 years subgroup **(E)** and the TyG index.

We conducted a RCS analysis to assess the potential non-linear relationship between the TyG index and stroke incidence (**Figure 5**). The P-value for non-linearity was 0.02, indicating a statistically significant non-linear relationship between TyG index levels and stroke risk in participants aged 60 years and older. A distinct U-shaped pattern was observed in this age group. Stroke risk initially decreased as the TyG index increased, up to a threshold of 8.47. However, beyond this threshold, stroke risk began to rise, becoming more pronounced at higher TyG index levels. This suggests that 8.47 may represent a critical inflection point in the relationship between TyG index and stroke risk in older adults.

In contrast, no significant non-linear relationships were observed between the TyG index and overall stroke, ischemic stroke, stroke in females, or stroke in individuals aged below 60 years.

## Discussion

This is the first studies to explore the predictive value of the TyG index for stroke and CVD in a low-income rural Chinese population. This 10-year prospective cohort study investigated the predictive value of the TyG index for stroke incidence in a low-income Chinese population, focusing on gender and age-specific differences. Both the value of TyG and its tertile grouping were confirmed to be independent predictors of stroke and ischemic stroke. Notedly, the association was not found in hemorrhagic stroke. Moreover, the TyG index was a stronger predictor of stroke in women and older adults (≥60 years). However, the relationship between TyG index and CVD risk was not found in this study.

The relationship between the TyG index and the incidence of different types of stroke has been previously reported in various studies. These studies have generally found that the TyG index is a significant predictor of ischemic stroke, but not hemorrhagic stroke. Several notable studies have highlighted the role of the TyG index in predicting stroke risk. For instance, a study by Wang et al. found that higher TyG index levels were associated with an increased risk of both total and ischemic stroke in a general Chinese population, but no significant association was observed with hemorrhagic stroke (15). Similarly, Hoshino et al. demonstrated that the TyG index is a valuable prognostic marker for major adverse cardiovascular events, including ischemic stroke, in Japanese patients with a history of ischemic stroke or transient ischemic attack (TIA) (16). Moreover, a meta-analysis confirmed that elevated TyG index levels were linked to a higher risk of cerebrovascular diseases, particularly ischemic stroke, across various populations (9). In another cohort study observed that the TyG index was a significant predictor of incident ischemic stroke, especially in older adults, but did not find a similar association with hemorrhagic stroke (17). Finally, the Kailuan study reported that long-term increases in the TyG index were associated with an elevated risk of ischemic stroke in a hypertensive population, while no significant link was found with hemorrhagic stroke (10). Consistent to these previous studies, our findings demonstrated that the TyG index is an independent predictor of total and ischemic stroke incidence, but not hemorrhagic stroke, in a low-income Chinese population. The potential mechanisms underlying these associations can be attributed to the role of IR in vascular health. Elevated TyG index levels indicate higher IR, which leads to oxidative stress, chronic inflammation, endothelial dysfunction, and accelerated atherosclerosis—all contributing factors to ischemic stroke (18–22). IR also increases thrombotic potential through enhanced platelet activity and impaired fibrinolysis, further exacerbating the risk of ischemic stroke (23–25). The lack of association with hemorrhagic stroke may be due to different pathophysiological processes involved, as hemorrhagic stroke is more related to factors such as hypertension and vascular integrity rather than metabolic disturbances like IR.

The relationship between the TyG index and the incidence of different types of stroke, particularly its stronger predictive value in women, has been reported in previous studies. Several studies have highlighted gender-specific differences in the association between the TyG index and stroke risk. The Rural Chinese Cohort Study found that the TyG index was significantly associated with an increased risk of ischemic stroke in women, but not in men, after adjusting for traditional risk factors (17). Similarly, a study reported that higher TyG index levels were more strongly associated with the risk of ischemic stroke in women compared to men in a large Chinese cohort (15). Furthermore, Zhao et al. observed that the TyG index was a significant predictor of stroke, particularly ischemic stroke, in women, with no significant association found in men (17). Additionally, a meta-analysis supported these findings, indicating that the TyG index was a more potent predictor of cerebrovascular events in women than in men (9). However, the Kailuan study showed a positive association between the TyG index and ischemic stroke in hypertensive men, but not in women, suggesting possible differences in study populations and comorbid conditions (10). Consistent to these previous studies, our findings also demonstrate that the TyG index is a stronger predictor of stroke, particularly ischemic stroke, in women. Several potential mechanisms may explain why the TyG index is a stronger predictor of stroke in women. Women generally have higher body fat percentages and different fat distribution patterns compared to men, which may influence IR and its metabolic consequences (18). Hormonal differences, particularly the protective effects of estrogen on vascular function and lipid metabolism, may also play a role. Post-menopausal women, who experience a decline in estrogen levels, may be more susceptible to IR and its associated vascular risks, thereby amplifying the predictive value of the TyG index for stroke (19, 20). Additionally, women may have different lifestyle factors, such as dietary habits and physical activity levels, that interact with metabolic risk factors differently than men (21, 22).

Several studies have highlighted age-specific differences in the association between the TyG index and stroke risk. TyG index was significantly associated with an increased risk of ischemic stroke in older adults, particularly those aged 60 years and above, in a large Chinese cohort (17), higher TyG index levels were more strongly associated with the risk of ischemic stroke in older adults compared to younger individuals (15), elevated TyG index levels were linked to a higher risk of cerebrovascular diseases, particularly in older populations (9). The Kailuan study showed that long-term increases in the TyG index were associated with an elevated risk of ischemic stroke in older adults with hypertension (10). Similar association was found in this study. Age-related increases in IR are well-documented, and older adults often exhibit higher levels of IR due to changes in body composition, decreased physical activity, and altered metabolic function (18, 19). These factors contribute to an increased risk of atherosclerosis and other vascular complications, which are major risk factors for ischemic stroke. Additionally, the cumulative exposure to cardiovascular risk factors over time may exacerbate the impact of IR on vascular health in older adults, making the TyG index a more potent predictor of stroke in this age group (20, 21). Furthermore, age-related endothelial dysfunction and increased arterial stiffness, both of which are associated with IR, may further enhance the risk of ischemic stroke in older individuals (22). These potential mechanisms may explain why the TyG index is a stronger predictor of stroke in older adults.

The association between the TyG index and the incidence of CVD has been well-documented in several studies, which generally indicate that the TyG index is a significant predictor of CVD. For instance, a cohort study by Laura Sánchez-Íñigo et al. found that the TyG index could predict the development of cardiovascular events (6). Similarly, the Kailuan study, which considered longitudinal changes in the TyG index over time, showed that cumulative TyG index was associated with an increased risk of CVD (8). Additionally, the PURE study, which examined populations in urban and rural areas across five continents, found that the TyG index was significantly associated with myocardial infarction and cardiovascular mortality (5). However, in our study, no significant association was observed between the TyG index and CVD incidence, including coronary atherosclerotic heart disease, myocardial infarction, and cardiovascular death. A potential explanation for this discrepancy is the absence of uniform coronary angiography in our cohort, which may have led to missed diagnoses of new coronary heart disease, myocardial infarction, and cardiovascular death, thereby affecting the accuracy of our findings.

This study has several limitations that need to be considered when interpreting the results. First, the study population was comprised of middle-aged and elderly individuals from low-income rural areas in northern China, which may limit the generalizability of our findings to other regions and socioeconomic groups. While this population is representative of other low-income rural populations in China, the specific environmental, lifestyle, and genetic factors in different regions may influence the association between the TyG index and stroke risk. The findings may not be directly applicable to urban populations or those with different socioeconomic backgrounds. Future studies should include diverse populations from various regions and socioeconomic statuses to enhance the generalizability of the results. Second, we assessed the predictive effect of the baseline TyG index on stroke incidence. Although this approach provides valuable insights into the initial risk assessment, it does not account for changes in the TyG index over time. IR and associated metabolic parameters can fluctuate, potentially altering stroke risk. The lack of multiple measurements over time may lead to an underestimation or overestimation of the true association between the TyG index and stroke risk. Therefore, future research should incorporate longitudinal measurements of the TyG index to capture its dynamic changes and better reflect its long-term impact on stroke incidence. Third, this study focused exclusively on first-time stroke events and did not investigate the relationship between the TyG index and recurrent strokes. Secondary stroke prevention is a critical aspect of managing patients who have already experienced a stroke, and understanding the role of the TyG index in predicting recurrent stroke risk is essential. By not including recurrent stroke events, we may have overlooked important information that could further elucidate the role of the TyG index in stroke prevention. Future studies should follow stroke survivors to explore the association between the TyG index and recurrent stroke risk, thereby providing a more comprehensive understanding of its utility in secondary prevention. Fourth, the absence of longitudinal data on the TyG index and the management of cardiovascular (CVS) risk factors throughout the 10-year follow-up period is a significant limitation. Only baseline measurements of the TyG index, TG, and FBG were collected, and no data were obtained on how these risk factors were managed over time, such as whether participants received treatment or achieved adequate control. This lack of longitudinal data hinders our ability to fully assess the dynamic relationship between the TyG index and stroke risk. For example, it remains unclear whether participants with high baseline TyG index levels improved their risk profiles through medication or lifestyle changes, potentially influencing long-term stroke outcomes. Consequently, this may lead to either an underestimation or overestimation of the true association between the TyG index and stroke risk, as the baseline measurement may not accurately capture the participant’s CVS risk over the 10-year period. Future studies should incorporate repeated measurements of the TyG index and other CVS risk factors to better capture how changes in these parameters over time affect stroke risk. Additionally, tracking the management of CVS risk factors, including medication use and lifestyle interventions, would provide valuable insights into the long-term impact of these factors on stroke outcomes. Furthermore, we did not collect data on whether participants with elevated triglyceride or fasting glucose levels received treatment or achieved optimal control during the follow-up period. This limitation restricts our ability to evaluate the effectiveness of managing CVS risk factors in individuals with high TyG index values, and whether such management mitigated stroke risk. Without information on the management of CVS risk factors, it is difficult to determine whether a high TyG index predicts poor control of these factors (e.g., persistent hyperglycemia or dyslipidemia) and consequently an increased risk of stroke. Participants with high baseline TyG index who received appropriate treatment may have reduced their risk, while those who remained untreated could have experienced worse outcomes. This lack of data introduces uncertainty into our interpretation of the predictive value of the TyG index. Future research should include detailed data on the treatment and control of CVS risk factors, including medication adherence and lifestyle modifications. Such data would provide a more comprehensive understanding of how managing these factors influences the relationship between the TyG index and stroke risk over time. Finally, potential confounding factors such as diet, physical activity, and medication use were not thoroughly controlled for in this study. These factors can significantly influence IR and stroke risk, and their omission may lead to residual confounding. Although we adjusted for several major confounders in our analysis, more detailed data on lifestyle factors and comprehensive adjustments are needed in future research to minimize potential biases and provide more accurate estimates of the TyG index’s predictive value.

## Conclusion

In this 10-year prospective cohort study, we demonstrated that the TyG index is an independent predictor of both total and ischemic stroke incidence in a low-income Chinese population. Our findings revealed that the TyG index was particularly effective in predicting stroke risk among women and older adults (≥60 years), but not hemorrhagic stroke. These findings highlight the potential of the TyG index as a valuable tool for stroke risk assessment. The TyG index, as a simple, cost-effective, and non-invasive measure of IR, can be readily integrated into routine clinical practice. Its use can facilitate early identification of individuals at high risk for ischemic stroke, allowing for timely intervention and personalized prevention strategies. Given its particular predictive value in women and older adults, the TyG index can aid clinicians in targeting these high-risk groups more effectively. Especially, the TyG index’s ability to predict stroke risk in a low-income rural population highlights its potential utility in resource-limited settings. By adopting the TyG index, healthcare providers in these areas can improve stroke prevention efforts and reduce the burden of stroke in vulnerable populations.

## Data Availability

The raw data supporting the conclusions of this article will be made available by the authors, without undue reservation.
