# Supplementary material for: Triglyceride-glucose index prediction of stroke incidence risk in low-income Chinese population: a 10-year prospective cohort study
Source: Front Endocrinol (Lausanne). 2024 Oct 17;15:1444030. doi: 10.3389/fendo.2024.1444030 (PMC11528446; doi:10.3389/fendo.2024.1444030)
Supplement: Supplementary file 1 [file Table1.docx]

Supplementary Table S1. The associated factors of cardiovascular diseases onset in univariate analysis

| Characteristic | Cardiovascular diseases | | |
| --- | --- | --- | --- |
|  | No | Yes | P |
| Case, n (%) | 3504 (99.1) | 31 (0.9) |  |
| Gender, n (%) |  |  | 0.026 |
| Men | 1400 (98.7) | 19 (1.3) |  |
| Women | 2102 (99.4) | 13 (0.6) |  |
| Age^*^, years | 59.00 (51.70, 65.66) | 62.19 (58.48, 67.13) | 0.013 |
| Age groups, n (%) |  |  | 0.014 |
| 45-59 years | 1911 (99.3) | 14 (0.7) |  |
| 60-74 years | 1315 (99.2) | 11 (0.8) |  |
| ≥75 years | 276 (97.5) | 7 (2.5) |  |
| BMI groups, n (%) |  |  | 0.513 |
| Normal | 1216 (99.3) | 9 (0.7) |  |
| Overweight | 1473 (99.1) | 13 (0.9) |  |
| Obesity | 807 (98.8) | 10 (1.2) |  |
| Smoking status, n (%) |  |  | 0.308 |
| Current smoking | 732 (99.3) | 5 (0.7) |  |
| Quit smoking | 149 (98.0) | 3 (2.0) |  |
| Never smoked | 2621 (99.1) | 24 (0.9) |  |
| Alcohol consumption, n (%) |  |  | 0.035 |
| Current drinking | 508 (99.6) | 2 (0.4) |  |
| Quit drinking | 38 (95.0) | 2 (5.0) |  |
| Never drank | 2956 (99.1) | 28 (0.9) |  |
| Hypertension, n (%) |  |  | 0.268 |
| Yes | 2417 (99.0) | 25 (1.0) |  |
| No | 1084 (99.4) | 7 (0.6) |  |
| Diabetes, n (%) |  |  | 0.676 |
| Yes | 664 (99.0) | 7 (1.0) |  |
| No | 2838 (99.1) | 25 (0.9) |  |
| SBP^*^，mmHg | 143.67 (130.17, 160.00) | 149.75 (137.75, 160.88) | 0.124 |
| DBP^*^，mmHg | 86.00 (79.00, 93.67) | 88.25 (77.50, 93.50) | 0.739 |
| Hb^*^, g/L | 138.00 (129.00, 271.00) | 134.00 (126.00, 142.00) | 0.153 |
| Plt^*^, 10^9^/L | 231.00 (197.00, 271.00) | 241.00 (210.00, 278.00) | 0.286 |
| FPG^*^, mmol/L | 5.60 (5.10, 6.10) | 5.85 (5.33, 6.34) | 0.041 |
| TC^*^, mmol/L | 4.79 (4.15, 5.50) | 4.95 (4.17, 5.49) | 0.618 |
| TG^*^, mmol/L | 1.40 (1.01, 2.10) | 1.50 (1.00, 2.61) | 0.401 |
| HDL-C^*^, mmol/L | 1.39 (1.15, 1.70) | 1.27 (1.10, 1.51) | 0.032 |
| LDL-C^*^, mmol/L | 2.59 (2.04, 3.21) | 3.00 (1.99, 3.50) | 0.177 |
| TyG index^*^ | 8.76 (8.39, 9.22) | 8.86 (8.44, 9.47) | 0.202 |
| TyG index tertile groups，n (%) |  |  | 0.666 |
| Tertile 1 | 1167 (99.2) | 10 (0.8) |  |
| Tertile 2 | 1169 (99.2) | 9 (0.8) |  |
| Tertile 3 | 1166 (98.9) | 13 (1.1) |  |

^*^Continuous variables were expressed as medians (percentile25, percentile75). TyG index, triglyceride-glucose index; BMI, body mass index; SBP, systolic blood pressure; DBP, diastolic blood pressure; Hb, hemoglobin; Plt, platelet; FPG, fasting plasma glucose;TG, triglycerides; TC, total cholesterol; LDL-C, low-density lipoprotein cholesterol; HDL-C, high-density lipoprotein cholesterol.
