# Supplementary material for: Triglyceride-glucose index prediction of stroke incidence risk in low-income Chinese population: a 10-year prospective cohort study
Source: Front Endocrinol (Lausanne). 2024 Oct 17;15:1444030. doi: 10.3389/fendo.2024.1444030 (PMC11528446; doi:10.3389/fendo.2024.1444030)
Supplement: Supplementary file 2 [file Table2.docx]

Supplementary Table S2. The associated factors of stroke onset in the sex subgroups in univariate analysis

| Characteristic | Stroke (in Male Subgroup) | | |  | Stroke (in Female Subgroup) | | |
| --- | --- | --- | --- | --- | --- | --- | --- |
|  | No | Yes | P |  | No | Yes | P |
| Case, n (%) | 1231 (86.8) | 188 (13.2) |  |  | 1931 (91.5) | 180 (8.5) |  |
| Age^*^, years | 59.65 (52.17, 66.75) | 63.31 (58.53, 70.97) | <0.001 |  | 57.77 (51.08, 63.95) | 63.08 (57.54, 69.29) | <0.001 |
| Age groups, n (%) |  |  | <0.001 |  |  |  | <0.001 |
| 45-59 years | 639 (91.3) | 61 (8.7) |  |  | 1161 (94.8) | 64 (5.2) |  |
| 60-74 years | 482 (83.4) | 96 (16.6) |  |  | 648 (86.6) | 100 (13.4) |  |
| ≥75 years | 110 (78.0) | 31 (22.0) |  |  | 126 (88.7) | 16 (11.3) |  |
| BMI groups, n (%) |  |  | 0.347 |  |  |  | 0.043 |
| Normal | 489 (88.3) | 65 (11.7) |  |  | 625 (93.1) | 46 (6.9) |  |
| Overweight | 498 (86.2) | 80 (13.8) |  |  | 832 (91.6) | 76 (8.4) |  |
| Obesity | 242 (84.9) | 43 (15.1) |  |  | 474 (89.1) | 58 (10.9) |  |
| Smoking status, n (%) |  |  | 0.259 |  |  |  | 1.000 |
| Current smoking | 596 (85.5) | 101 (14.5) |  |  | 37 (92.5) | 3 (7.5) |  |
| Quit smoking | 125 (85.6) | 21 (14.4) |  |  | 6 (100) | 0 (0) |  |
| Never smoked | 510 (88.5) | 66 (11.5) |  |  | 1892 (91.4) | 177 (8.6) |  |
| Alcohol consumption, n (%) |  |  | 0.305 |  |  |  | 0.740 |
| Current drinking | 423 (86.9) | 64 (13.1) |  |  | 22 (95.7) | 1 (4.3) |  |
| Quit drinking | 37 (94.9) | 2 (5.1) |  |  | 1 (100) | 0 (0) |  |
| Never drank | 771 (86.3) | 122 (13.7) |  |  | 1912 (91.4) | 179 (8.6) |  |
| Hypertension, n (%) |  |  | <0.001 |  |  |  | <0.001 |
| Yes | 795 (82.7) | 166 (17.3) |  |  | 1316 (88.9) | 165 (11.1) |  |
| No | 436 (95.2) | 22 (4.8) |  |  | 618 (97.6) | 15 (2.4) |  |
| Diabetes, n (%) |  |  | <0.001 |  |  |  | <0.001 |
| Yes | 1043 (88.5) | 135 (11.5) |  |  | 362 (84.2) | 68 (15.8) |  |
| No | 188 (78.0) | 53 (22.0) |  |  | 1573 (93.4) | 112 (6.6) |  |
| SBP^*^，mmHg | 144.00 (130.50, 158.33) | 154.50 (143.00, 172.63) | <0.001 |  | 141.50 (128.00, 158.50) | 155.50 (141.00, 174.58) | <0.001 |
| DBP^*^，mmHg | 87.00 (80.50, 94.50) | 91.83 (83.63, 100.50) | <0.001 |  | 84.50 (77.50, 92.00) | 90.05 (82.50, 97.00) | <0.001 |
| Hb^*^, g/L | 138.00 (130.00, 148.00) | 139.00 (131.00, 147.25) | 0.681 |  | 137.00 (129.00, 147.00) | 138.00 (129.00, 148.00) | 0.443 |
| Plt^*^, 10^9^/L | 231.00 (197.00, 273.00) | 221.00 (198.75, 254.50) | 0.171 |  | 232.00 (196.00, 272.25) | 226.00 (200.00, 266.00) | 0.839 |
| FPG^*^, mmol/L | 5.60 (5.20, 6.10) | 5.70 (5.30, 6.49) | 0.016 |  | 5.50 (5.10, 6.10) | 5.90 (5.40, 6.90) | <0.001 |
| TC^*^, mmol/L | 4.50 (3.86, 5.19) | 4.76 (4.06, 5.37) | 0.010 |  | 4.94 (4.30, 5.65) | 5.26 (4.65, 6.06) | <0.001 |
| TG^*^, mmol/L | 1.24 (0.92, 1.89) | 1.25 (0.91, 1.88) | 0.831 |  | 1.51 (1.08, 2.21) | 1.78 (1.26, 2.57) | 0.001 |
| HDL-C^*^, mmol/L | 1.32 (1.10, 1.61) | 1.34 (1.08, 1.62) | 0.790 |  | 1.43 (1.20, 1.74) | 1.42 (1.19, 1.72) | 0.786 |
| LDL-C^*^, mmol/L | 2.44 (1.95, 3.03) | 2.63 (2.12, 3.19) | 0.009 |  | 2.66 (2.10, 3.29) | 2.83 (2.26, 3.47) | 0.009 |
| TyG index^*^ | 8.63 (8.28, 9.10) | 8.71 (8.28, 9.11) | 0.466 |  | 8.81 (8.45, 9.26) | 9.13 (8.67. 9.57) | 0.004 |
| TyG index tertile groups，n (%) |  |  | 0.530 |  |  |  | <0.001 |
| Tertile 1 | 503 (87.8) | 70 (12.2) |  |  | 569 (94.2) | 35 (5.8) |  |
| Tertile 2 | 398 (86.7) | 61 (13.3) |  |  | 675 (93.9) | 44 (6.1) |  |
| Tertile 3 | 330 (85.3) | 57 (14.7) |  |  | 691 (87.2) | 101 (12.8) |  |

^*^Continuous variables were expressed as medians (percentile25, percentile75). TyG index, triglyceride-glucose index; BMI, body mass index; SBP, systolic blood pressure; DBP, diastolic blood pressure; Hb, hemoglobin; Plt, platelet; FPG, fasting plasma glucose;TG, triglycerides; TC, total cholesterol; LDL-C, low-density lipoprotein cholesterol; HDL-C, high-density lipoprotein cholesterol.
