# Supplementary material for: Triglyceride-glucose index prediction of stroke incidence risk in low-income Chinese population: a 10-year prospective cohort study
Source: Front Endocrinol (Lausanne). 2024 Oct 17;15:1444030. doi: 10.3389/fendo.2024.1444030 (PMC11528446; doi:10.3389/fendo.2024.1444030)
Supplement: Supplementary file 3 [file Table3.docx]

Supplementary Table S3. The associated factors of stroke onset in the age subgroups in univariate analysis

| Characteristic | Stroke (in <60 years Subgroup) | | |  | Stroke (in ≥60 years Subgroup) | | |
| --- | --- | --- | --- | --- | --- | --- | --- |
|  | No | Yes | P |  | No | Yes | P |
| Case, n (%) | 1800 (93.5) | 125 (6.5) |  |  | 1366 (84.9) | 243 (15.1) |  |
| Gender, n (%) |  |  | 0.003 |  |  |  | 0.010 |
| Men | 639 (91.3) | 61 (8.7) |  |  | 592 (82.3) | 127 (17.7) |  |
| Women | 1161 (94.8) | 64 (5.2) |  |  | 774 (87.0) | 116 (13.0) |  |
| BMI groups, n (%) |  |  | 0.007 |  |  |  | 0.079 |
| Normal | 550 (95.0) | 29 (5.0) |  |  | 564 (87.3) | 82 (12.7) |  |
| Overweight | 786 (94.2) | 48 (5.8) |  |  | 544 (83.4) | 108 (16.6) |  |
| Obesity | 462 (90.6) | 48 (9.4) |  |  | 255 (82.8) | 53 (17.2) |  |
| Smoking status, n (%) |  |  | <0.001 |  |  |  | 0.158 |
| Current smoking | 373 (88.4) | 49 (11.6) |  |  | 260 (82.5) | 55 (17.5) |  |
| Quit smoking | 67 (93.1) | 5 (6.9) |  |  | 64 (80.0) | 16 (20.0) |  |
| Never smoked | 1360 (95.0) | 71 (5.0) |  |  | 1042 (85.8) | 172 (14.2) |  |
| Alcohol consumption, n (%) |  |  | 0.011 |  |  |  | 0.640 |
| Current drinking | 262 (89.7) | 30 (10.3) |  |  | 183 (83.9) | 35 (16.1) |  |
| Quit drinking | 17 (100) | 0 (0) |  |  | 21 (91.3) | 2 (8.7) |  |
| Never drank | 1521 (94.1) | 95 (5.9) |  |  | 1162 (84.9) | 206 (15.1) |  |
| Hypertension, n (%) |  |  | <0.001 |  |  |  | <0.001 |
| Yes | 1100 (90.5) | 116 (9.5) |  |  | 1011 (82.5) | 215 (17.5) |  |
| No | 700 (98.7) | 9 (1.3) |  |  | 354 (92.7) | 28 (7.3) |  |
| Diabetes, n (%) |  |  | <0.001 |  |  |  | <0.001 |
| Yes | 278 (86.1) | 45 (13.9) |  |  | 272 (78.2) | 76 (21.8) |  |
| No | 1522 (95.0) | 80 (5.0) |  |  | 1094 (86.8) | 167 (13.2) |  |
| SBP^*^，mmHg | 138.00 (126.00, 151.50) | 151.50 (137.67, 167.17) | <0.001 |  | 149.00 (135.42, 165.50) | 159.00 (143.00, 175.67) | <0.001 |
| DBP^*^，mmHg | 86.00 (79.46, 93.50) | 93.00 (86.50, 101.00) | <0.001 |  | 84.50 (77.50, 92.50) | 90.50 (82.00, 98.50) | <0.001 |
| Hb^*^, g/L | 138.00 (130.00, 147.00) | 138.00 (128.00, 146.25) | 0.955 |  | 137.00 (128.00, 147.00) | 138.00 (130.75, 148.25) | 0.189 |
| Plt^*^, 10^9^/L | 232.00 (196.00, 272.00) | 240.50 (199.50, 281.25) | 0.527 |  | 230.00 (197.00, 273.00) | 220.00 (200.00, 252.00) | 0.085 |
| FPG^*^, mmol/L | 5.49 (5.10, 6.00) | 5.80 (5.40, 6.80) | <0.001 |  | 5.60 (5.20, 6.20) | 5.70 (5.30, 6.70) | 0.004 |
| TC^*^, mmol/L | 4.73 (4.07, 5.42) | 4.92 (4.21, 5.73) | 0.042 |  | 4.84 (4.23, 5.56) | 4.95 (4.39, 5.78) | 0.018 |
| TG^*^, mmol/L | 1.43 (1.02, 2.17) | 1.52 (1.09, 2.39) | 0.189 |  | 1.34 (0.99, 1.99) | 1.51 (0.96, 2.12) | 0.163 |
| HDL-C^*^, mmol/L | 1.37 (1.15, 1.66) | 1.32 (1.12, 1.59) | 0.185 |  | 1.41 (1.16, 1.77) | 1.41 (1.14, 1.72) | 0.721 |
| LDL-C^*^, mmol/L | 2.53 (2.00, 3.15) | 2.62 (2.10, 3.39) | 0.109 |  | 2.63 (2.05, 3.24) | 2.75 (2.22, 3.35) | 0.039 |
| TyG index^*^ | 8.77 (8.38, 9.23) | 8.93 (8.50, 9.50) | 0.004 |  | 8.71 (8.38, 9.15) | 8.86 (8.37, 9.34) | 0.014 |
| TyG index tertile groups，n (%) |  |  | 0.021 |  |  |  | 0.003 |
| Tertile 1 | 589 (95.0) | 31 (5.0) |  |  | 483 (86.7) | 74 (13.3) |  |
| Tertile 2 | 606 (94.2) | 37 (5.8) |  |  | 467 (87.3) | 68 (12.7) |  |
| Tertile 3 | 605 (91.4) | 57 (8.6) |  |  | 416 (80.5) | 101 (19.5) |  |

^*^Continuous variables were expressed as medians (percentile25, percentile75). TyG index, triglyceride-glucose index; BMI, body mass index; SBP, systolic blood pressure; DBP, diastolic blood pressure; Hb, hemoglobin; Plt, platelet; FPG, fasting plasma glucose;TG, triglycerides; TC, total cholesterol; LDL-C, low-density lipoprotein cholesterol; HDL-C, high-density lipoprotein cholesterol.
